# Effect of Fasting on Adrenal Insufficiency During Ramadan: Patients’ Knowledge, Attitude, and Practice

**DOI:** 10.7759/cureus.97468

**Published:** 2025-11-21

**Authors:** Fatimah F Altaleb, Amal Alturkistani, Marwah Bafadel, Nouf Aboalsamh, Mussa H Almalki

**Affiliations:** 1 Obesity, Endocrine, and Metabolism Center, King Fahad Medical City, Second Health Cluster, Riyadh, SAU; 2 Alfaisal University, College of Medicine, Riyadh, SAU

**Keywords:** adrenal insufficiency, fasting, patient’s knowledge, ramadan, steroid

## Abstract

Background

Adrenal insufficiency (AI), a condition stemming from adrenal hormone deficiency (primarily cortisol), significantly impacts bodily functions. Ramadan fasting, involving 12-18 hours of daily abstinence, presents serious challenges for individuals with AI, potentially leading to severe health complications. The understanding of AI and its challenges among Saudi Arabian patients remains under-researched.

Objective

This study assessed the knowledge, attitudes, and fasting practices of AI patients in Saudi Arabia during Ramadan.

Methods

A cross-sectional survey was conducted (October 15-December 15, 2023) at a tertiary hospital's endocrine outpatient clinic. Patients with AI aged (≥14 years) completed a 33-item structured questionnaire evaluating their understanding of AI, Ramadan fasting practices, and relevant demographic and clinical data.

Results

Among the 158 participants (predominantly female, 87 (55.1%), and aged 14-20 years, 49 (31%) ), 65 (41.1%) lacked knowledge of AI's primary causes, and 75 (47.5%) felt inadequately informed despite receiving healthcare. While 140 (88.6%) fasted (135 (85.4%) with physician approval), 68 (43%) hadn't discussed fasting plans with providers. Furthermore, 132 (83.5%) were unaware of fasting's implications for their condition, and 75 (47.5%) experienced fatigue. Treatment adjustments were made for 89 (56.3%) during Ramadan, yet many felt unprepared.

Conclusion

This study reveals substantial knowledge gaps among Saudi Arabian AI patients regarding Ramadan fasting, increasing health risks. Despite widespread willingness to fast, inadequate patient information highlights the urgent need for improved education, provider support, and structured interventions to ensure safe fasting practices.

## Introduction

Adrenal insufficiency (AI) is a medical condition characterized by deficient production of adrenal hormones, primarily cortisol and aldosterone, which regulate critical physiological processes such as blood pressure homeostasis, glucose metabolism, immune modulation, and stress response [1]. AI is classified as primary (due to adrenal gland dysfunction, e.g., Addison’s disease), secondary (due to pituitary insufficiency), or tertiary (due to hypothalamic dysfunction) [2]. Common etiologies include autoimmune disorders, infections (e.g., tuberculosis), genetic mutations, and prolonged glucocorticoid therapy [3,4]. Symptoms range from chronic fatigue, weight loss, and orthostatic hypotension to life-threatening adrenal crises marked by hypoglycemia, hypotension, and shock [5]. During the holy month of Ramadan, Muslims abstain from food, fluids, and medications from dawn to sunset (typically 12-18 hours daily). Fasting imposes significant metabolic and physiological stress, particularly for individuals with endocrine disorders like AI [6]. Cortisol, a key hormone in glucose regulation and stress adaptation, is critical during fasting to maintain energy homeostasis and cardiovascular stability. Patients with AI lack this adaptive response, increasing their susceptibility to dehydration, hypoglycemia, electrolyte imbalances, and adrenal crises during prolonged fasting [7,8]. Despite these risks, studies suggest that many patients with AI attempt to fast during Ramadan, often without adequate medical guidance [9]. In Saudi Arabia, the prevalence of AI remains undetermined, though clinical reports indicate it is underrecognized [10,11]. This knowledge gap complicates risk stratification and patient education. Moreover, variability in cortisol assay performance may contribute to diagnostic uncertainty in AI, potentially affecting clinical decisions [12]. Existing literature highlights inconsistent practices in managing AI during Ramadan, with limited consensus on glucocorticoid dose adjustments, hydration strategies, or criteria for exempting high-risk patients from fasting [13,14], reflecting common challenges encountered in endocrine care during periods of religious fasting. For instance, sudden changes in steroid dosing schedules or missed doses due to fasting windows may precipitate adrenal crises [5]. Current guidelines emphasize individualized care, recommending pre-Ramadan assessments to evaluate disease stability, educate patients on symptom recognition, and adjust medication timings. However, implementation remains inconsistent due to cultural and religious motivations to fast, coupled with limited provider-patient communication [11,13]. Regional data from Saudi Arabia are scarce. Furthermore, the role of healthcare providers in guiding safe fasting practices-such as splitting corticosteroid doses, ensuring adequate salt intake, and using sick-day rules-requires further exploration [15,16].

Objective

This study aims to evaluate the knowledge, attitudes, and practices of AI patients in Saudi Arabia regarding fasting during Ramadan. By identifying gaps in patient education and clinical management, this research seeks to inform context-specific guidelines to mitigate complications and improve health outcomes.

## Materials and methods

Design 

This cross-sectional study utilized a structured questionnaire and was conducted at King Fahad Medical City (KFMC), a tertiary care hospital, between October 15, 2023, and December 15, 2023. The study population included all patients (aged ≥14 years) diagnosed with adrenal AI, who were under regular follow-up at the adult endocrinology clinics. Exclusion criteria comprised individuals with contraindications to fasting (e.g., acute illness, recent travel, pregnancy, or lactation) based on religious exemptions, as well as children under 14 years of age. Eligible participants were identified through electronic medical records and recruited consecutively during routine outpatient endocrine clinic visits. The study team approached potential participants in person, explained the study purpose and procedures, and enrolled those who met the inclusion criteria and agreed to participate. Informed consent was obtained from all eligible participants prior to questionnaire administration. The study protocol received ethical approval from the Institutional Review Board (IRB) of the Research Center at KFMC (Reference Number: [23‐ 293]).

Questionnaire 

The questionnaire was developed de novo by two authors (M.A. and F.A.) and revised by the remaining authors to align with the study’s primary objectives. Participants completed a 33-item questionnaire (Appendices: Table 6) assessing their knowledge of AI and fasting practices during Ramadan. Clinical data, including AI etiology, disease duration, comorbid hormonal deficits, and concurrent conditions, were collected. Prior to implementation, the questionnaire underwent rigorous validation through both expert panel review (evaluating content relevance, statistical validity, and design adequacy) and a pilot study involving 20 AI patients to test comprehensibility and reliability. Internal consistency was confirmed via Cronbach’s alpha (α=0.853), demonstrating excellent reliability.

Statistical analysis

Descriptive statistics summarized demographic and clinical characteristics. Continuous data were expressed as mean ± SD, and categorical variables as percentages. Chi-square tests assessed associations between variables (p < 0.05). Analyses were performed using SPSS v29 (IBM Corporation, Armonk, NY, USA).

## Results

A total of 158 participants were included in the study, with their sociodemographic characteristics summarized in Table 1. Participants ranged in age from 14 to over 50 years, with the largest proportion, 49 (31.0%), in the 14-20-year age group. Females comprised 87 (55.1%) of the sample, and males accounted for 71 (44.9%). The duration of AI was evenly distributed, with 50 (31.6%) of participants having the condition for over 15 years.

**Table 1:** Sociodemographic Characteristics of Participants (n = 158)

Parameter		No.	Percent (%)
Age	14-20 years	49	31.0
	21-30 years	10	6.3
	31-40 years	42	26.6
	41-50 years	26	16.5
	More than 50 years	31	19.6
Gender	Female	87	55.1
	Male	71	44.9
Duration of adrenal insufficiency	Less than 1 year	14	8.9
	1-5 years	45	28.5
	6-10 years	36	22.8
	11-15 years	13	8.2
	More than 15 years	50	31.6

As shown in Table 2, participants demonstrated significant gaps in knowledge regarding adrenal insufficiency. A total of 65 (41.1%) were unaware of the primary cause of their condition, while 47 (29.7%) attributed it to pituitary disease and 46 (29.1%) to adrenal gland disease. Nearly half of the participants, 75 (47.5%), reported insufficient knowledge about their condition.

**Table 2:** Parameters Related to Participants’ Knowledge of the Effect of Fasting on Adrenal Insufficiency (n = 158)

Parameter		No.	Percent (%)
What is the primary cause of adrenal insufficiency?	As a result of a disease in the pituitary gland	47	29.7
	As a result of a disease in the adrenal gland itself	46	29.1
	I don’t know	65	41.1
Do you have adequate information about adrenal insufficiency?	No	75	47.5
	Yes	83	52.5
Does your doctor explain and clarify the treatment for your condition?	No	8	5.1
	Yes	150	94.9
Do you carry an emergency card indicating adrenal insufficiency?	No	126	79.7
Have you been provided with steroid injections for emergencies?	No	120	75.9
	Yes	38	24.1
Is adrenal insufficiency a chronic disease?	No	30	19.0
	Yes	128	81.0
Does adrenal insufficiency require lifelong treatment?	No	16	10.1
	Yes	142	89.9
What treatment are you currently using for adrenal insufficiency?	Hydrocortisone	104	65.8
	Hydrocortisone and Fludrocortisone	39	24.7
	Prednisone	8	5.1
	Dexamethasone	2	1.3
	Fludrocortisone	2	1.3
	Fludrocortisone/Dexamethasone	1	.6
	Cortisone, I don’t know what kind	1	.6
	Prednisolone and Fludrocortisone	1	.6
	How often do you take steroid treatment in a day?	Once daily	30	19.0
Twice daily		110	69.6
Three times daily		13	8.2
Do not take it regularly		5	3.2
What is the timing of your steroid treatment?	Morning	19	12.0
	Evening	9	5.7
	One dose in the morning and one in the evening	124	78.5
	There is no specific time	6	3.8
Do you increase your steroid dose when necessary (e.g., during illness or health issues)?	No	23	14.6
	Yes	135	85.4
How many times per year do you visit the emergency room due to adrenal insufficiency?	Once	59	37.3
	Twice	21	13.3
	Three times	5	3.2
	Four times	11	7.0
	More than five times	1	.6
	I don't know	2	1.3
	None	59	37.3

Despite these gaps, 150 (94.9%) confirmed that their physicians adequately explained treatment options. However, only 32 (20.3%) carried an emergency card for AI, and 120 (75.9%) lacked emergency steroid injections. Hydrocortisone was the most common treatment, 104 (65.8%), followed by combined hydrocortisone and fludrocortisone, 39 (24.7%). Most participants, 110 (69.6%), took steroids twice daily, with 124 (78.5%) adhering to morning and evening dosing. Additionally, 135 (85.4%) reported increasing steroid doses during illness.

**Figure 1:**
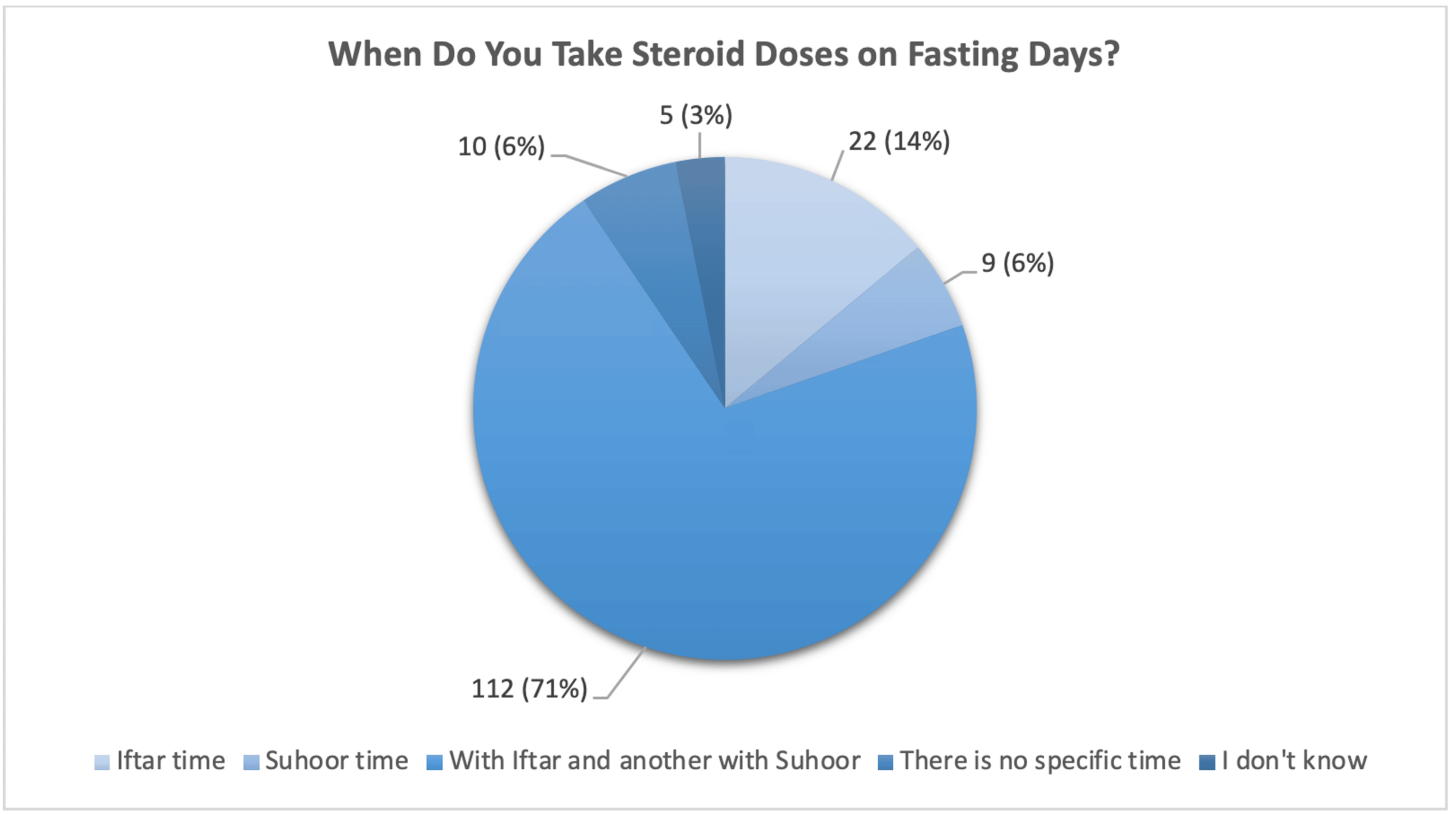
Distribution of Steroid Dose Timing During Fasting Among Participants

Regarding fasting practices, 140 (88.6%) fasted during Ramadan, yet 98 (62.0%) received no specific guidance for managing their condition. Although 128 (81.0%) recognized AI as a chronic disease requiring lifelong treatment, 132 (83.5%) were unaware of fasting’s impact. Steroid dosing schedules included concurrent administration at Iftar and Suhoor 112 (70.9%), Iftar-only 22 (13.9%), Suhoor-only 9 (5.7%), and irregular timing 10 (6.3%) (Figure 1).

Symptoms during fasting were reported by 75 (47.5%), with lethargy 27, 17.1%) and fatigue 24, 15.2%) being the most common. While 64 (40.5%) broke their fast immediately upon experiencing symptoms, 94 (59.5%) continued fasting. Most participants, 127 (80.4%), completed the fasting month, supported by hydration, 135 (85.4%), and therapeutic adjustments, 89 (56.3%) (Table 3).

**Table 3:** Participants’ Attitudes and Practices Toward the Effect of Fasting on Adrenal Insufficiency (n = 158)

Parameter		No.	Percent (%)
Do you consume foods with a balanced amount of salt?	No	34	21.5
	Yes	124	78.5
Do you attend regular clinic visits?	No	25	15.8
	Yes	133	84.2
Do you fast during Ramadan?	No	18	11.4
	Yes	140	88.6
Have you discussed fasting during Ramadan with your doctor?	No	68	43.0
	Yes	90	57.0
Doctor allowing you to fast during Ramadan?	No	23	14.6
	Yes	135	85.4
Would you like to know more about fasting during Ramadan?	No	33	20.9
	Yes	125	79.1
Were you provided with instructions or guidelines for managing adrenal insufficiency during Ramadan?	No	98	62.0
	Yes	60	38.0
Do you know how fasting affects your condition?	No	132	83.5
	Yes	26	16.5
Do you feel tired, fatigued, or experience any symptoms while fasting?	No	83	52.5
	Yes	75	47.5
If yes, what symptoms did you experience while fasting?	Fatigue	24	15.2
	Headache	8	5.0
	Shaking or trembling	3	1.9
	lethargy	27	17.1
	Unwillingness to move	5	3.2
	Numbness	1	0.6
	Dizziness	4	2.5
	Extreme thirst	3	1.9
	Nausea and Abdominal pain	3	1.9
	Hypoglycemia	4	2.5
	None	83	52.5
If you feel tired or have symptoms, do you immediately break your fast?	No	94	59.5
	Yes	64	40.5
If you break your fast, do you resume fasting the following day?	No	37	23.4
	Yes	121	76.6
Do you feel capable of fasting?	No	19	12.0
	Yes	139	88.0
Did you fast for the entire month?	No	31	19.6
	Yes	127	80.4
Do you drink plenty of fluids?	No	23	14.6
	Yes	135	85.4
Did your doctor adjust your treatment schedule or dosage during Ramadan?	No	69	43.7
	Yes	89	56.3
When do you take steroid doses on fasting days?	Iftar time	22	13.9
	Suhoor time	9	5.7
	With Iftar and another with Suhoor	112	70.9
	There is no specific time	10	6.3
	I don't know	5	3.2
Did you require an emergency room visit for an adrenal insufficiency crisis during Ramadan?	No	139	88.0
	Yes	19	12.0
If yes, how many times did you visit the emergency room?	None	139	88.0
	Four times	1	0.6
	Three times	1	0.6
	Twice	5	3.1
	Once	12	7.6

No significant associations were found between gender (p = 0.678) or age (p = 0.438) and knowledge adequacy (Table 4).

**Table 4:** Relationship Between Having Adequate Information About Adrenal Insufficiency and Sociodemographic Characteristics *A p-value ≤ 0.05 was considered statistically significant.

Parameters		Do you have enough information about adrenal insufficiency?		Total (N=158)	P-value*
		No	Yes		
Gender	Female	40	47	87	0.678
		53.3%	56.6%	55.1%	
	Male	35	36	71	
		46.7%	43.4%	44.9%	
Age	14-20 years	22	27	49	0.438
		29.3%	32.5%	31.0%	
	21-30 years	3	7	10	
		4.0%	8.4%	6.3%	
	31-40 years	23	19	42	
		30.7%	22.9%	26.6%	
	41-50 years	10	16	26	
		13.3%	19.3%	16.5%	
	More than 50 years	17	14	31	
		22.7%	16.9%	19.6%	
Duration of adrenal insufficiency?	Less than 1 year	26	19	45	0.157
		34.7%	22.9%	28.5%	
	1-5 years	6	7	13	
		8.0%	8.4%	8.2%	
	6-10 years	20	16	36	
		26.7%	19.3%	22.8%	
	11-15 years	17	33	50	
		22.7%	39.8%	31.6%	
	More than 15 years	6	8	14	
		8.0%	9.6%	8.9%	

However, younger participants (14-20 years) demonstrated greater awareness of fasting implications (p = 0.022). Disease duration showed no association with understanding fasting effects (p = 0.516) (Table 5).

**Table 5:** Relationship Between Fasting and Illness Among Participants According to Sociodemographic Characteristics * A p-value ≤ 0.05 was considered statistically significant.

Parameters		Do You Know How Fasting Affects Your Illness?		Total (N=158)	P-value*
		No	Yes		
Gender	Female	70	17	87	0.247
		53.0%	65.4%	55.1%	
	Male	62	9	71	
		47.0%	34.6%	44.9%	
Age	14-20 years	22	27	49	0.022
		29.3%	32.5%	31.0%	
	21-30 years	3	7	10	
		4.0%	8.4%	6.3%	
	31-40 years	23	19	42	
		30.7%	22.9%	26.6%	
	41-50 years	10	16	26	
		13.3%	19.3%	16.5%	
	More than 50 years	17	14	31	
		22.7%	16.9%	19.6%	
Duration of adrenal insufficiency?	Less than 1 year	11	3	14	0.516
		8.3%	11.5%	8.9%	
	1-5 years	41	4	45	
		31.1%	15.4%	28.5%	
	6-10 years	11	2	13	
		8.3%	7.7%	8.2%	
	11-15 years	30	6	36	
		22.7%	23.1%	22.8%	
	More than 15 years	39	11	50	
		29.5%	42.3%	31.6%	

Regarding healthcare utilization during Ramadan, only 19 (12.0%) required emergency room visits during Ramadan, with 12 (7.6%) visiting once and 5 (3.1%) twice. Additionally, healthcare providers adjusted treatment regimens for 89 (56.3%) of participants, while 69 (43.7%) received no modifications.

## Discussion

This study explored the knowledge, attitudes, and practices regarding fasting in patients with AI, highlighting significant gaps in understanding that could impact health outcomes. The majority of participants were young adults, reflecting the demographic profile of AI patients, with a notable female predominance of 87 (55.1%), consistent with literature suggesting a higher prevalence of AI in women [17]. The findings reveal critical knowledge deficits: 65 (41.1%) of participants were uncertain about the etiology of their condition, and 75 (47.5%) felt inadequately informed, consistent with previous reports highlighting limited awareness among patients with adrenal insufficiency [18]. This underscores the urgent need for improved patient education, particularly regarding the physiological implications of fasting. While 150 (94.9%) reported that their physicians explained treatment options, discussions rarely addressed lifestyle factors such as fasting, indicating a gap in comprehensive care. Fasting during Ramadan poses unique challenges for AI patients due to the risks of dehydration, hypoglycemia, and adrenal crises [7,8]. Despite these risks, 140 (88.6%) of participants fasted, yet 132 (83.5%) were unaware of how fasting affects their condition. Alarmingly, 126 (79.7%) did not carry emergency medical identifiers, and 120 (75.9%) lacked emergency glucocorticoid injections-a finding mirroring global reports of suboptimal crisis preparedness in AI populations [17,19]. In regions like Saudi Arabia, where fasting is culturally entrenched, these gaps are particularly concerning due to inconsistent clinical guidance [11].

Symptoms such as fatigue and lethargy were reported by 75 (47.5%) of participants during fasting, yet 94 (59.5%) continued fasting despite discomfort. This aligns with Chihaoui et al., who reported that 67% of fasting AI patients experienced complications, even when advised against fasting [9]. The prioritization of religious observance over health, compounded by inadequate provider-patient communication (68 (43%) did not discuss fasting plans with clinicians), emphasizes the need for culturally sensitive interventions [13]. Interestingly, younger participants (aged 14-20 years) demonstrated marginally better understanding of fasting implications (p = 0.022), though overall knowledge remained insufficient. This contrasts with Reisch et al., who linked younger age to poorer adherence to stress dosing in AI, suggesting that age-related educational strategies may differ [19]. The pharmacokinetics of glucocorticoid replacement further complicate fasting. Cortisol’s circadian rhythm - peaking at dawn and declining by dusk - necessitates tailored dosing during Ramadan, when oral intake is restricted to nighttime [20]. While 112 (70.9%) adjusted doses to Iftar (sunset) and Suhoor (predawn), 10 (6.3%) reported erratic timing (6.3%) and 22 (13.9%) used single-dose regimens, both of which risk under-replacement, exacerbating hypoglycemia and hypotension [5]. Although 89 (56.3%) had treatment adjustments during Ramadan, 69 (43.7%) received no modifications, reflecting inconsistent clinical management. Cultural factors play a pivotal role: 135 (85.4% ) fasted despite knowledge gaps, highlighting societal pressures prevalent in Muslim-majority countries [13]. Structured interventions, such as pre-Ramadan workshops and emergency injection training, could bridge this gap. For instance, Beshyah et al. demonstrated that patient education reduced hypoglycemia rates by 40% in fasting diabetics - a model adaptable to AI care [13]. This study has limitations. Its single-center design may limit generalizability, and self-reported data introduce recall bias. Additionally, the exclusion of acutely ill patients may underestimate complication rates. Future research should explore longitudinal outcomes and the efficacy of targeted educational programs.

## Conclusions

While many patients with AI are motivated to observe fasting practices during Ramadan, their insufficient understanding of the associated health risks remains a significant concern. Healthcare providers must focus on comprehensive education regarding the implications of fasting, individual management strategies, and foster ongoing communication to ensure patient safety. Future studies should evaluate the effectiveness of tailored educational interventions on patient outcomes, ultimately striving to cultivate a knowledgeable and health-conscious patient population.

## Appendices

**Table 6:** Adrenal Insufficiency Patient Questionnaire

No.	Question	Response Options
1	Consent	I agree to participate; I do not agree to participate
2	Age	14-20 years; 31-40 years; 41-50 years; More than 50 years
3	Gender	Male; Female
4	Duration of adrenal insufficiency	Less than one year; 1-5 years; 6-10 years; 11-15 years; More than 15 years
5	Cause of adrenal insufficiency	Adrenal gland disease; Pituitary gland disease; I don't know
6	Do you have enough information about adrenal insufficiency?	Yes; No
7	Has your doctor explained the treatment?	Yes; No
8	Do you carry an emergency card?	Yes; No
9	Do you have emergency steroid injections?	Yes; No
10	Is adrenal insufficiency a chronic disease?	Yes; No
11	Does adrenal insufficiency require lifelong treatment?	Yes; No
12	Current treatment	Hydrocortisone; Fludrocortisone; Hydrocortisone and Fludrocortisone; Prednisone; Other
13	Frequency of steroid treatment	Once daily; Twice daily; No regular treatment; Other
14	Timing of steroid doses	Morning; Evening; Morning and Evening; No specific time
15	Do you increase steroid dose when sick?	Yes; No
16	ER visits per year due to adrenal crisis	Once; Twice; Three times; Four times; Other
17	Do you eat a balanced amount of salt?	Yes; No
18	Do you visit the clinic regularly?	Yes; No
19	Do you fast during Ramadan?	Yes; No
20	Have you discussed fasting with your doctor?	Yes; No
21	Has your doctor allowed fasting?	Yes; No
22	Do you want more information about fasting?	Yes; No
23	Have you received Ramadan guidance?	Yes; No
24	Do you know how fasting affects your condition?	Yes; No
25	Do you feel symptoms during fasting?	Yes; No
If yes	Describe symptoms and timing	Your answer:
26	Do you break your fast when symptomatic?	Yes; No
27	Do you resume fasting the next day?	Yes; No
28	Do you feel able to fast?	Yes; No
29	Do you fast the whole month?	Yes; No
30	Do you drink plenty of fluids?	Yes; No
31	Did your doctor change medication during Ramadan?	Yes; No
32	When Do You Take Steroid Doses on Fasting Days?	At Iftar; At Suhoor; Both Iftar and Suhoor; No specific time; I don't know
33	Did you go to ER during Ramadan?	Yes; No
If yes	How many times?	Your answer:
